# "The Hospital" Nursing Mirror

**Published:** 1900-10-13

**Authors:** 


"The Hospital" Nursing Mirror.

Being the Nursing Section of "The Hospital."

[Contributions for this Section of "The Hospital" should be addressed to the Editor, "The Hospital" Nursing Mirror, 28 & 29 Southampton Street, Strand, London, W.C.]

### Notes on News from the Nursing World.

### NURSES AND THE PLAGUE.

The Matron of Belvidere Hospital, Glasgow, stated in her Notes on Nursing the Plague that there was "a ready response for volunteers to nurse in the plague wards." Unfortunately, a case has occurred at Cardiff. The patient was removed to the Fever Hospital, and immediately on his arrival a nurse, with characteristic courage, volunteered to attend him. Of course, there are risks, but, as the account of nursing at the Maratha Plague Hospital, Bombay, now appearing in our columns shows, they are not so great as some people imagine.

### NURSES ON THE "KILDONAN CASTLE."

The passengers on board the "Kildonan Castle," which has just arrived with a number of sick and wounded men from South Africa, included three members of the Army Nursing Service Reserve, namely Misses E. Cruickshank, L. D. Hill, and A. N. Bowles. The nurses on duty were Sisters B. F. Jones and F. M. Williams. The feature of the voyage was an outbreak of small-pox, which, happily, did not spread beyond four men, who sickened with the disease two days after the vessel left the Cape, so effective were the precautions taken for their isolation.

### SISTERS AND CONVALESCENT SOLDIERS.

"An Alexandra Nurse" writes from Maritzburg:—

"At the invitation of Lady Tullibardine, who is a guest of Sir W. Hely Hutchison, some 200 of the convalescents have just been entertained to tea and music at Government House. I had not an opportunity of being present, but the Tommies said, 'It only lacked one thing to make it perfect, the presence of the sisters.' Finding I was not the recipient of a ticket, one of my patients thrust his into my hand with 'Sister, do go, the men will like it so much better!' The day was perfect and all enjoyed it. The men said that Captain Meiklejohn, V.C., waited upon them 'just as if we had been gentlemen.' On learning that the first hospital train with men on board who had been wounded at the battle near Krugersdorp and Belfast was to pass through our station, one or two ladies, deeply interested in the soldiers, met it on its arrival, 6.20 a.m. They distributed jerseys, mufflers, caps, Tam o' Shanters, handkerchiefs and literature, &c., while cigarettes and tobacco were not forgotten. I accompanied the ladies on their labour of love, and it was a pleasure to hear from the men that the long railway journey —they had come straight through from Pretoria—had been made so comfortable for them. On account of the unsettled state of the country they had only travelled by daylight, but this also gave greater opportunity for a better rest and sleep. As the curves are very sharp and numerous on this line, the oscillation is also very considerable, and most trying even to those in perfect health, but I never heard a grumble anywhere."

### A POSSIBLE DANGER.

A Matron of one of the London hospitals tells us she is rather afraid that the nurses who went to South Africa from civilian hospitals may find the ordinary routine of ward work somewhat tame by comparison with their exciting experiences at the Cape and elsewhere. We know that more than one nursing sister has owned to a feeling of restlessness on her return to England. It may be hoped, however, that the mere fact of having nursed under difficulties and different conditions will, after the restlessness has worn off, have the practical effect of making nurses more resourceful in emergency, and more capable of exercising the faculty of common-sense.

### PATIENTS ON THE "PERSIA."

The writer of the article with this heading in our last number wishes to correct a mistake that occurred through the omission of a word in the account of the invalids on the s.s. "Persia," which she thinks might easily wound the feelings of the relatives of the two men who died at sea. She continues: "The sentence ran: 'No one expected they would recover,' and this makes it appear as if, the case of the men being hopeless, it mattered little whether they were brought away or not. As a matter of fact, what the Kilburn sister said was: 'No one expected they would *not* recover,' meaning that it was a surprise to the P.M.O. and the nursing staff that they died. It will be noticed that no cases thought to be at all serious were brought any further than Cape Town, and this being so, the two who died should apparently have been put on shore there. But they were considered quite convalescent enough to undertake the voyage, and in the case of one at least 'he looked so well when he came on board' (said the sister) 'that we did not recognise him until he told us he had been a patient of ours at Ladysmith.' Both died from sudden failure of the heart, being convalescent from pneumonia."

### "'TOMMIES' BY THE SEA."

The matron and nurse of the Fletcher Convalescent Home, Cromer, have several invalided "Tommies" from South Africa now under their care. From Netley and Aldershot these men have been sent to see what the bracing air of Cromer will do for them. Otherwise, this admirable home is solely for the benefit of convalescents from the Norfolk and Norwich Hospital.

### AN EXPERIMENT BY A QUEEN'S NURSE.

It may interest district nurses to hear of an experiment made by a Queen's nurse in a country district, which has so far been very successful. For some time she lived in lodgings, but last year a gentleman, having two small cottages standing in a nice garden, offered to put them in good repair and let them to the nurse for 2s. 9d. a week each. The idea was, that the nurse should live in one, and a woman in the other, who, in return for rent and firing, should cook, clean, and wait on the nurse. This is what has been done. The furniture was partly bought and given, and partly lent. As the cottage only contains four small rooms, the front door opening direct into the sitting-room, a great deal of furniture was not needed. The red-tiled kitchen is used as a dining-room, all the cooking being done next door; the wash-house serves as coal-cellar and bicycle house. A pound a week, with 10s. monthly extra in winter for firing, is allowed for household expenses, and this proves quite sufficient. The association pay no more than they did for the lodgings, whereas the nurse now possesses two sitting-rooms, and even a little spare bedroom, to say nothing of a garden, which produces sufficient fruit and vegetables for her use. Of course the comfort of this arrangement depends entirely on the woman engaged to do the work. In this instance she has no children, and a very quiet, well-behaved husband. There is no communication between the two cottages, which is an advantage for the nurse.

### VISITING NURSES FOR THE MIDDLE CLASSES.

THE remarks of Mrs. Dacre Craven on visiting nurses for the middle classes, reported in an interview in the MIRROR last week, have attracted a good deal of attention. A correspondent whose letter appears in another column puts in a special plea for the working ladies who live in big towns, daily governesses, secretaries, type-writers and others, who usually live in two rooms, or in small flats. We agree with "One of the Public" that when they are ill their condition is one of great wretchedness. Even more than families whose means are limited, they seem to need caring for; though visiting nurses are often badly wanted, as Mrs. Craven showed, in homes where there is not sufficient accommodation to keep an extra person in the home. Of course, the question of fees is one of vital importance. Working ladies find it extremely difficult to earn guineas, and it is absolutely impossible for them to pay for nursing at the rate of 3s. 6d. an hour. We suspect that a solution of the difficulty is only to be found in nurses following the example of doctors and taking the rough with the smooth, adapting their charges according to the circumstances of the patients.

### THE INDUSTRIAL LAW COMMITTEE AND NURSES.

AN interesting debate will take place on Friday, the 19th, at the Midwives' Institute, 12 Buckingham Street, Strand, at 8 p.m., on "Industrial Laws as they concern Nurses." The speakers will be Mrs. Talbot, wife of the Bishop of Rochester, and Miss de Chaumont, Sanitary Inspector for Kensington, on behalf of the Industrial Law Committee—an organisation which is doing excellent work in spreading abroad a wider knowledge of the laws existing for the protection of life and health, more especially as they affect women and children. The heads of nursing institutions are specially invited to the lecture, as it is hoped through their co-operation and interest to reach those working under them—particularly district nurses, to whom a practical knowledge of the protection given by the law to the health of women and children while at work is of great value. Amongst the many workers who can materially help in this matter, no one, perhaps, has such good opportunities as the nurse. She has, as one of the leaflets issued by the Committee states, "the absolute confidence of her patients; she is with them in the times of their greatest suffering—suffering not seldom caused by some accident or incident of their occupation"—and in her hands rests much preventive power. Nurses who wish for further information should apply to Miss Boileau, Secretary, at the offices of the Committee, 5 Palmer Street, Westminster.

### AGRICULTURAL LABOURERS AND NURSING.

WHEN the Waltham Nursing Association was started more than two years ago, it was decided to ask the agricultural labourers for a subscription of two shillings per year for the privilege of having the use of a nurse in their family should they require her, and in the event of her services being needed to pay a very small sum for her work. At a bazaar the other day in aid of the funds of the association, Mr. Alderman Doughty stated that this system had answered exceedingly well; and Lord Yarborough, who said he was struck by the fact that the labourers were called upon to subscribe, expressed his approval of the principle, on the ground that it had the very desirable effect of doing away with the idea that the association was a charity. He also considered that it was likely to make the labourers take a great interest in the nurse and her work. This is a sound view. People usually value more what they have to pay for, however small the amount may be, and we believe that the most sure way of securing the trained nurse a welcome in the cottages of the agricultural labourers is to send her there, not as the agent of a charity, but as the minister of relief to whose services they feel they have a right.

### THE GUILDFORD GUARDIANS AND THE NURSING REGULATIONS.

THE result of the inquiry of the House Committee of the Guildford Board of Guardians into the relations existing between the various officers of the workhouse is that the rules in force for the nursing staff have been amended. The report of the committee was discussed at some length at the fortnightly meeting of the Board. The substance of it was as follows:—

> In accordance with the resolution passed by the Guardians at their meeting held on August 11th, the committee interviewed all the officers of the workhouse (with the exception of the matron, who was absent on leave), and report that the relations between them appear to be generally satisfactory, but that there seems to be some slight friction existing between the master and the superintendent nurse, and a coolness between him and the two day nurses. The committee therefore recommend (1) That the superintendent nurse shall have power to grant applications for extra leave of absence from the other nurses, provided that the duties of the applicant can be properly carried out during her absence; such extra leave to be only for short periods, and for special reasons, and in every case to be notified immediately to the master; that in the case of the annual leave of the superintendent nurse, she should apply to the Board through the clerk. (2) That the applications from the nurses and assistant nurses for their annual leave be made to the Board through the clerk, such applications to be countersigned by the superintendent nurse.

The adoption of the report was opposed by some of the Guardians, chiefly on the ground that they thought there should be no division of power, but the supporters of it contended that the superintendent nurse is the proper officer to judge when a nurse can be spared. Unquestionably the latter are right, and we are glad that the report was carried, though it remains to be seen whether, so long as there is want of harmony between the superintendent nurse and the master, friction can be avoided by any alterations in the nursing regulations.

### NO PROGRESS AT KENDAL.

AT a meeting of the Kendal Board of Guardians the Clerk reported that the Milnthorpe Workhouse Committee had considered the question of a trained nurse for that institution, but "were unanimously of opinion that there was no need for any change." This retrograde view was supported by the Guardians, who evidently have no wish to keep pace with the times. Now that it has at last been decided that a trained nurse may be a necessity in a convict prison infirmary, the refusal to provide a workhouse infirmary with one is all the more to be regretted.

### A HANDSOME GIFT TO THE TAUNTON NURSING ASSOCIATION.

ON the recommendation of the Committee of the District Nursing Association, the Taunton Diamond Jubilee Trustees, who have a fund of £736 in hand for the acquisition of a Nurse's Home, had decided to purchase Canon Lodge, in Canon Street. The position is central, and the building is admirably constructed for the purpose. But the negotiations for purchase came suddenly to an end, owing to an intimation from the owner—Mr. George

Saunders, of Lydeard House, Bishop's Lydeard—that he wished to present the house to the Association, in memory of his late wife and father and mother. The generosity of Mr. Saunders will enable the Association to devote part of the sum they possess to the provision of furniture for the Home, while the balance will form the nucleus of an endowment. Miss Fisher, who lately resigned the post of Lady Superintendent, will continue to act as honorary secretary of the Association, and her services will be available in making all the arrangements in connection with the Home.

### DISTRICT NURSING IN THE ISLE OF WIGHT.

Like Newport, Ryde and Cowes have admirable district nursing associations, which are both doing excellent work in the Isle of Wight. At the annual meeting of the Ryde Association it was stated that the nurses paid upwards of 7,000 visits in the year and had 300 new cases. The rules are those of Queen Victoria's Jubilee Institute, and, as Dr. Davey said at the meeting, "they are a sufficient guarantee that the institution is carried out on lines that are workable." Another speaker, the Vicar of Ryde, complimented the nurses on the tact and patience they had shown in getting their instructions carried out—testimony which was not the less appreciated because the uncle of the vicar's wife, the late Mr. W. S. Broadwood, left all his money to a nurse. At Cowes 2,569 visits were paid by the nurse to 115 patients, and the committee of the Nursing Association are anxious it should be clearly understood that she is, as she should be, at the call of the sick poor belonging to any religious denomination and in all parts of the town.

### A RASH INVESTMENT.

A strange story was told in the London Sheriffs' Court last week, in the case of an action for the recovery of damages for breach of agreement. The plaintiff, Mrs. Annie Garraud, a widow, has for many years been a certificated hospital nurse. In 1890 she went to South Africa, and, according to the statement of her counsel and her own evidence, was superintendent of the hospital at Barberton. For her services in connection with the construction of the Beira Railway she was presented by Mr. Cecil Rhodes with a farm. This year she was invalided home, and took a house at Northampton. Wanting something to do, she answered an advertisement and eventually purchased from a Mr. Smith, for £200, a house in Marylebone, described as "a splendid hotel property." But when Mrs. Garraud proceeded to take possession she found that it was a house of disrepute, and at once very properly shut it up. The defendant did not appear at the trial, and the jury awarded the plaintiff £250 damages. We hope that she will get them, but her imprudence in buying a "splendid hotel property" without any knowledge of it was certainly amazing. She may have been an admirable nurse, but hardly a good woman of business.

### LEITER'S TUBE.

When going round the "Woman's Exhibition" at Earl's Court for the last time two nurses were much amused by the remarks of those who came to view the Charing Cross exhibits and the "Field Hospital." They noticed particularly one young couple who were evidently "doing their honeymooning." They were of the 'Arry and 'Arriet type: she had him firmly by the arm, and took him round from one bed to another, explaining as best she could the different appliances and their uses, until they came to the last bed. The supposed patient, who lay there staring with glassy eyes, puzzled her for a while; then a brilliant solution of the mystery occurred to her. Showing her husband the "coil" which surrounded the unfortunate one's skull, she said: "See there, ain't that handy?—that's to *let the gas go round 'is 'ed.*" "Ay, ain't it clever?" was his admiring response.

### THE CHILDREN'S HOSPITAL, SYDNEY.

The city of Sydney, New South Wales, has been in need of a children's hospital for many years. The building used for the purpose at present was not built for it, being several private houses utilised for want of better accommodation. In this, however, the needs of the children have been most ably attended to by a devoted staff of doctors and nurses, who, in spite of every adverse circumstance, have coped with the always increasing strain on income and accommodation. Again and again distracted parents have been turned away from the inadequate institution to seek treatment and admission elsewhere; the delay and exposure often aggravating the malady of the patient until treatment became useless, especially in the case of diphtheria. Now, at last, a movement on a large scale has been started; influential people have taken the matter up, and help is coming from all directions. A site for a new building has been granted in one of the many beautiful parks with which Sydney abounds; a strong committee has been formed, and money is not only being given freely, but is being raised by all kinds of alluring entertainments. The friends of the little sick ones are rejoicing already, for the success of the scheme is fairly assured; and soon a beautiful children's hospital, worthy of such a generous people and such a prosperous city, will be added to its host of charities.

### AMERICAN NURSES IN CHINA.

According to the New York *Trained Nurse* of October, eleven American nurses have been retained by General Chaffee for duty in China, while five others originally intended for Manila are already at work in the Flowery Land. Our contemporary says:—"In all probability these are the only women nurses with the allies on Chinese soil, and their position is, therefore, one of extreme interest. Serving at the front with an army in a hostile country and among uncivilised peoples is something of a novelty for trained nurses, and our British cousins will probably lament greatly their absence from the same scene."

### SHORT ITEMS.

The Totnes Board of Guardians have decided not to institute an enquiry into the cause of the nurses leaving. The committee who reported upon the matter stated that "the superintendent nurse did not wish to go into particulars."—The committee appointed by the United Friendly Societies in connection with the Wisbech Town nursing scheme have decided to continue to pay £10 per year to the funds of the Wisbech and District Nursing Association for another term of three years.—The experiment of sending probationers to be trained at Plaistow is being made by the Technical Instruction Committee of the Flintshire County Council, not of the Cheshire County Council, as a correspondent unintentionally led us to infer.

### Lectures on Nursing for Probationers.

By E. MACDOWEL COSGRAVE, M.D., Lecturer to the Dublin Metropolitan Technical School for Nurses.

### No. XVII.—WATER.

WATER is necessary for life, for it enters into all the tissues of the body, and forms the greater part of the secretions and of the blood. Indeed, about three-quarters of the body is made up of water.

Water is usually a liquid, but in cold weather it becomes converted into solid ice; in doing so it expands, so ice is lighter than water and floats. It is this expansion which bursts pipes during frost; but as the water does not escape until the thaw, the popular idea is that the thaw breaks the pipes. When water is boiled it gradually loses its liquid form and passes into vapour.

When a water supply is being arranged for a town, at least 30 gallons a head per day are allowed. This seems a great deal, but only 3½ to 5 pints are required for actual consumption: part of this is taken in the form of beverages, and part in food. The rest of the supply is required for cooking, washing, bathing, flushing drains, &c.

A scanty supply means uncleanliness of house, utensils, clothes and person, unflushed drains and contaminated air.

Pure water has neither colour, smell nor taste, and shows no suspended matter; if kept for a couple of days in a cool, clean place it ought to remain unchanged.

Water is made up of two gases—oxygen and hydrogen—and is formed whenever these two gases are burned together; this can be seen by holding a clean plate for a moment or two in a gas flame, when drops of moisture will be observed to form upon it.

The sources of drinking water are:—

Rain,
Rivers,
Lakes,
Springs,
Sea.

All come from the same source, as without rain all the rest would dry up. When rain falls on the earth part goes back to the air in the form of vapour, part flows over the surface of the ground into rivers and lakes, and part sinks into the ground, reappearing in wells and springs. Most of the rain comes from the vapour given off by the sea, and the sea is replenished by the rivers.

Rain that falls through clear air is practically as pure as distilled water when it reaches the earth; when, however, it passes through the smoke-laden, breath-contaminated atmosphere of a city it picks up many impurities. Rain water is generally collected from roofs into tanks or barrels: a good deal of dust, organic matter, &c., is washed down with it, and so rain water stored for use is always contaminated. Rain water is extremely soft, and acts on lead, so if stored in a lead cistern, or drawn through lead pipes, it is dangerous.

River water is supplied chiefly by the rain water, which either runs directly off the surface or else soaks into the ground, flowing into the river from springs. River water is therefore contaminated by manure and other decaying matter being washed in; it is, however, most seriously contaminated by the drainage of villages and towns.

River water is generally well aërated, especially if the water ripples over shallows, but, owing to the amount of matter suspended in it, it needs filtering.

Lake water is much the same as river water, but does not contain so much sediment, as it has had time to settle: it is rather tasteless, as it is not well aërated.

Springs arise from rain which has sunk into the ground: in the ground it gets charged with carbonic acid gas, which enables it to dissolve lime. As the water soaks through the soil it becomes purified, organic matter being filtered out. Deep wells are safest, as it requires some depth of soil to purify water; they should be far from drains, and should have the upper part lined with brick to prevent insufficiently filtered water getting in, and should have a coping round the mouth to prevent heavy rain washing in surface impurities.

Sea water can be purified from salt by distillation, the vapour being condensed. Distilled water is tasteless from its softness and want of air; the latter can be supplied by pouring it backwards and forwards from one jug to another.

Hardness of water depends upon the presence of salts which decompose soap and prevent lathering until enough soap has been added to exhaust them. Hardness may be either temporary or permanent.

Temporary hardness is caused by carbonates; such water will wash, but will not lather: boiling removes temporary hardness, as does the addition of lime water. The latter method is used with good effect at some waterworks, and it is found that 1 cwt. of quicklime, costing 8d., softens the water so much that £47 worth of soap is saved.

Permanent hardness is caused by sulphates: such water will neither wash nor lather, and cannot be softened by boiling, or by the addition of lime.

Water may be contaminated by dead organic matter, or by living organisms: these latter multiply rapidly, especially if organic matter be present.

Organic contamination is shown by the presence of nitrites, or of salt. Salt suggests contamination by urine; nitrites show sewage contamination (except in peaty districts).

A simple test for salt is to add a few drops of dilute nitric acid to a tumbler of the water, and then to drop in a few drops of nitrate of silver solution: if salt is present there will be a white precipitate.

To test for organic impurity, acidulate a teacupful of the water with a little sulphuric acid, then add Condy's fluid until the water is of a rose colour; if the colour is soon discharged it shows the water is contaminated. It must be remembered that peaty water will have the same effect.

Contaminated water may cause diarrhœa, undermine the health and predispose to disease. Typhoid fever and cholera are chiefly spread by water contaminated by their germs. In hot climates dysentery may be caused in the same way.

Lead poisoning is also caused by water containing lead: the chief symptoms are dyspepsia, constipation, colic, paralysis of the extremities, and a blue line on the gums.

Distillation removes all impurities, whether dissolved or suspended.

Filtering also removes impurities, but great care must be taken that the filter is clean, otherwise it will only make the water more impure. Charcoal filters rapidly become foul. Pasteur's filter of unglazed porcelain is the best, but only water under high pressure will pass through it, and it is these high-pressure supplies that least need filtering.

The best treatment for doubtful water is to boil it; it should afterwards be aërated by pouring it from one jug to another.

Water should if possible never be stored, but should be drawn directly from the pipe: water kept in a cistern is liable to contamination by sewer gas.

Water should not be kept in a sick room, but brought in as required.

Thirst is best slaked by warm water, acidulated drinks such as lemonade and apple tea, and drinks such as barley water, which leave a moist coating on the tongue.

### Nursing at Winburg, Orange River Colony.

By an Army Sister.

It was almost with a sense of disappointment that three of us, Nursing-Sisters of the Army Reserve, received orders, about the middle of May, to proceed for duty to Winburg, in the Orange River Colony. "We shall be side-tracked and cut off from all excitement for the rest of the campaign," said the American member of our party, for we knew that the town was not on the main line, but situated at the end of the small branch of railway which leaves the trunk at Smaldeel. Contrary to all expectation, however, Winburg, which was the first capital of the State, has proved a most interesting station; it has been the base for the lengthy operations in the north-east of the colony against General de Wet, and must so continue, as long as the Boers persist in the useless guerilla warfare they are now waging. After a comfortable and interesting journey from Cape Town to Bloemfontein, fourteen hours' ride in the guard's van of a troop train of open trucks seemed but an adventure and experience of campaigning. The guard was a very good friend, did his best to convert the mail-bags into lounges, and pointed out the scenes of recent events.

### Appropriating the Officers' Dinner.

The four hours we had to spend at Smaldeel, waiting till the line was clear, would have been very trying but for our good fortune in straying into the messing quarters of the officers stationed there. Here was a table spread for dinner, and, being much too hungry to stop and consider whether this was a public or a private eating-house, we began our meal without further ado. Great was our consternation when those for whom it was intended put in their appearance. We attempted an explanation; but I need hardly say that they took it in very good part, even insisting that fortune was on their side. After dinner, the tale of their experiences in the field passed the time until we again renewed our journey.

### An Unexpected Arrival.

Our arrival in Winburg, soon after midnight, was certainly not timed at the happiest moment, nor did it appear that we were expected; indeed, the telegram announcing our departure from Bloemfontein did not arrive until twenty-four hours after we did—not at all an unusual occurrence it seems. The railway staff officer proposed a very bare waiting-room as a lodging, and the sight of a fire in the grate (for it was bitterly cold) made us decide to lie in our rugs on the floor. We all three slept very soundly. The next day (Sunday) we spent as a day of rest in a very literal sense, and we made ourselves fairly comfortable in a furnished house which had been commandeered for our use.

### Plenty of Hard Work.

Monday we began work, and hard work too. Enteric fever, then at its worst, was raging in the hospitals. Of these when we first came there were two besides the officers' hospital. Both the Town Hall and the Government School had been used for the purpose by the Boers throughout the campaign, and were in charge of the medical and nursing staffs sent by the Netherlands Red Cross Society, who, however, left with the Boers when they evacuated Winburg on the 6th May, the date of the British occupation. By the time we entered on our duties, these hospitals were only occupied by our soldiers, though a few wounded Boers were still being cared for by the townsfolk in another building.

### A Church Converted into a Hospital.

Nearly all our patients were suffering from typhoid. The Town Hall had beds for twenty-six, but the School was only furnished for ten, though forty men were accommodated there. The beds, of course, were given to the worst cases; but it was a sad sight to see many of the poor fellows lying on the floor in their clothes, one blanket doubled under them, and covered by another. Surgical cases at that time were conspicuous by their absence, but it soon became evident that further accommodation must be found for the victims of the epidemic, which continued on the increase. It is to the credit of all concerned—and help came from all sides—that as soon as this was apparent no time was lost in opening the Dutch Church, a temporary iron building, and fitting it as a hospital. In forty-eight hours the mud floor was boarded, beds and bedding (commandeered from shops and vacated houses) for fifty-six had been put in, each bed as it was put together being immediately occupied. Not only were there those from the Winburg garrison to provide for, but convoys of sick were continually arriving from the front and swelling our numbers.

### An Inevitable Hardship of War.

These journeys by convoy were of three or more days' duration, their speed never exceeding fourteen miles a day. Of the sufferings of the men racked with the pain of fever it is impossible to think calmly, and after the severe jolting to which they were subjected in the ox-waggons it seemed as if no effort could save some of these cases, and we lost many cases. We cried out at first at the cruelty of sending men in such a condition on such a journey; after a time we understood that it was one of the inevitable hardships of war. Many of these men had fallen out on the march; a moving column cannot be hampered by its sick, and by the first opportunity they must be carried to the base by convoy. From Senekal, although a hospital was established there, we had hundreds of patients; our troops there were hard pressed by De Wet's commando, food supplies were almost impossible to obtain, and hospital accommodation was very scarce. Here the ambulance trains could relieve the pressure whenever necessary.

### A Novel Surgical Hospital.

The Dutch Church being opened for severe cases, and other buildings used for convalescents, the School was available as a surgical hospital. The full equipment for an operating-room, as well as surgical appliances and dressings, had been left behind by the Dutch; and, indeed, for some weeks after our arrival we were all but dependent on their stores for drugs and foodstuffs. Winburg is one of those places where the transport difficulty is never lost sight of. In time, however, beds, sheets, and blankets for the School arrived from the base, and the hospital was in great demand after the battle and veld fire at Senekal. The patients did not come to us all at once, but as they were able to travel, fifty or sixty in a convoy, and it was many a long day before we saw the last trace of that dreadful fire.

### "Not a Nurse there."

It was at this period that we heard such bad accounts of the epidemic of typhoid at Senekal, not to mention the cases of severe burns and wounds. "Not a nurse there," they said, and so much needed. In vain we volunteered for duty; we knew we could easily be replaced from Bloemfontein. The regulations, we were told, do not permit nursing-sisters to leave the railhead. Why shoul l this be so if the nursing required is beyond that point?

### Boer Prisoners.

When over four thousand prisoners were brought here after General Prinsloo's surrender, we had a few Boer patients in hospital; they appeared to be worn out with the hardships they had endured. The two convoys which brought them to Winburg were a most interesting sight. The first was a procession five miles long, and took ten hours to come in. It is easy to understand this, for, except about four hundred, who were mounted, the prisoners rode in waggons drawn by a team of fourteen oxen each, and not carrying more than fifteen or sixteen men. There were, besides, the captured guns and a great number of horses. They were very soon drafted down country by rail, and are now doubtless comfortably settled in Ceylon. A fourth sister has lately joined us, and for some time now our work has been very much lighter. We have comparatively few cases of enteric; unfortunately, it is anticipated that there will be more as it grows hotter. The flies, too, are again becoming unbearable.

### An Amputation by Candle-light.

Whatever may have been our idea of nursing during a campaign before we left home 'most of us must now know that it seldoms happens that it is the duty of an English army sister to remove the field dressing. Two or three times, however, we have had the wounded brought straight from the field of action; this has happened when an outgoing convoy of supplies has been attacked outside the town. Upon one occasion, when Colonel Ridley, during a reconnaissance, was surrounded by overwhelming numbers at a farm ten miles from here, and held out for two days with some two hundred Colonials, our ambulances, which had been sent, returned in the night, and it was found necessary to do an amputation of the thigh at once, four or five candles being the only light available. Five killed and twenty-two wounded was the heavy price paid for the gallant stand. The funeral next day of those lost in action was a mournful scene.

### An Engagement at our Doors.

Two days after, on August 26th, the same Boers made an attack on Winburg from three sides. After three months of rumours and false alarms, it was difficult to believe that the firing we could hear so plainly was really the long-expected attack, but the sound of the Boer shells bursting was convincing. We had to go but a few paces from our doorstep to get a good view of the engagement. There were our men in trenches not 300 yards off. A mile away we could see our artillery getting into position and the infantry in extended order advancing towards the kopje which was the object of attack and was held by a few of the garrison. We noted with satisfaction that the Boers had not got the range of the kopje, and that their shells were bursting without doing any damage. We wondered if our guns were doing any better, for we could not see where the projectiles fell, although the Boers galloping towards the kopje were visible in the distance. Just then the guns, which were placed near the railway station, opened fire on a kopje in an opposite direction. It was held by the enemy, and from that point, we were afterwards told, they intended to enter the town, the other attack being a feint to draw off the artillery, but the Boers, finding they had very wrongly estimated the strength of Winburg, deemed it prudent to withdraw without loss of time, not, however, before General Olivier was taken prisoner, whether by design or not is uncertain. We saw him as he passed, under escort, to General Bruce-Hamilton's quarters. Our casualties that day, I am glad to say, did not number more than one killed and three wounded

### Important to all Trained Nurses.

### THE NURSE'S WHO'S WHO.

The forms to be filled up for the forthcoming edition of "*Burdett's Official Nursing Directory*" by the nurses who are not at present entered there, have now been sent out to all whose names and addresses have been obtainable, and those nurses who have not returned their form, or who have not yet made application for one to fill up, should do so at once to the Acting Editor, 28 and 29, Southampton Street, Strand, W.C., as October 24th is the latest date for receiving new names for the next issue.

As a result of the articles which appeared in the Nursing Mirror for January 13th and August 4th of this year, to which nurses who have not seen them should refer, a large number of forms have been applied for. This means either that the Directory had not previously been brought to the notice of these nurses, or that the advantages of having their names entered in it had not been thoroughly understood.

With regard to the nurses whose names are already in the Directory, the proofs for correction have been sent out, and the Acting Editor will be glad to have these returned with any necessary corrections and additions without delay. Any nurse whose name is in the Directory, and who has not received a proof during the last few days, should at once apply to the Acting Editor for it, as in the event of her having changed her address or taken up a new appointment the letter has very probably miscarried. This of course is unavoidable, so long as nurses neglect to give notice of change of address. A proportion of letters, happily less than in former years, are returned through the post marked "Not known" or "Left the hospital, present address unknown," the result being that the names of these nurses appear in the Directory with an asterisk against them, signifying that there is no guarantee that the particulars given are correct beyond a certain date. It is obvious that this is extremely detrimental to a nurse.

### Resignation of the Lady Superintendent of Melbourne Hospital.

The matron of Melbourne Hospital, Miss Farquharson has resigned her appointment. The circumstances are reported as follows in the *Melbourne Argus* of September 5: The Melbourne Hospital Committee met yesterday, Mr. J. Grice presiding. The special committee appointed to investigate the cause of a slight amount of friction which was said to exist between the lady superintendent, Miss Farquharson, and the nursing staff, presented a report, in which certain alterations in the arrangement of hours of duty and leave of absence were suggested. In presenting the report, Mr. J. S. Butters said that the committee had dealt exhaustively with the matter, but many of the nurses had left since the necessity for appointing a committee arose, and the relations between the matron and the present staff were much more harmonious. The report was referred to a sub-committee to deal with the recommendations, the chairman remarking that although there had apparently been complaints of undue strictness, the inquiry had proved Miss Farquharson to be a most satisfactory matron. Miss Farquharson wrote intimating that it was her intention to resign her position as lady superintendent on December 12th, and took the earliest opportunity of acquainting the committee, in order that arrangements might be made for the appointment of a successor. It was resolved that advertisements be circulated throughout Australia and New Zealand inviting applications for the position.

### The Nurse in Hot Climates.

By Edward Henderson, M.D., F.R.C.S. Edin., late Surgeon, General Hospital, Shanghai.

(*Continued from page 8.*)

*Voyage.*—When choice is possible, the voyage should be timed so that the nurse may reach the end of her journey after the hot weather—in the end of September or in October. The nurse's object is to arrive at her destination in as good health and as fit for work as possible; and to attain this end she must exercise care, and possibly a little self-denial, in regulating her life on board ship. She will naturally be careful as to what she eats and drinks, and should try to take all the active exercise the opportunities afforded permit. Since the introduction of refrigerating chambers on board passenger steamers the task of providing fresh food of every kind has been greatly simplified; but even under these improved conditions perfect safety cannot always be guaranteed, and on one or two occasions, towards the end of the voyage, travellers have noticed a peculiar tainting of certain articles of food preserved in this way, which suggested commencing decomposition; quite possibly the condition was developed under the influence of hot weather after the food had been removed from the refrigerator—and we know that frozen meat when thawed decomposes readily—but, however it was occasioned, and under whatever circumstances it arose, if suspected, it should at once warn the consumer to exercise increased care at table. An attack of diarrhœa, brought on by an indiscretion in diet, may yield readily enough to a dose of castor oil and a little enforced abstinence; but such an occurrence is to be regretted, and the sufferer, who is experiencing tropical weather for the first time, may find diarrhœa by no means such a simple matter as any previous experience she has had of the complaint in England might have led her to suppose. Special care should be taken by the traveller who goes on shore at any of the ports of call on the way, as the food supplied in some of the hotels she may visit is often of a doubtful character. Under such circumstances it is safer to drink bottled aërated water than plain water, and milk had better be avoided altogether. Fruit should always be eaten with care, for, though its effect as a cause of bowel disorders has been greatly exaggerated, real danger may attend its consumption if the necessary precautions have been neglected. Those who are familiar with Eastern bazaars know that the opened water-melons, in order to preserve their fresh appearance, are often sprinkled by the vendors with water of a distinctly dangerous kind; and much the same is true of many of the other fruits and vegetables exposed for sale. Apart from this practice, the attacks of flies, and the mere handling of the fruit by the dirty hands of the native salesman, may be sources of danger to the consumer. It is difficult to purify grapes by washing; and as in eating grapes the skins have to be sucked, they may be taken to represent a class of fruit at this time better avoided altogether. The thick rinds of the mangosteen, pummelo, and pineapple effectually protect the part eaten from contamination; while in the case of plantains, mangoes, and the like, ordinary care taken in removing the skins will generally be a sufficient precaution.

As a simple traveller, if not in her professional capacity, the nurse will no doubt hear a good deal from her fellow-passengers regarding sea-sickness and its treatment. Some favoured individuals are able, it would seem, to master the disease by a determined effort made from the first; but the number who succeed under really trying circumstances is small, and in such people it is doubtful whether the malady would ever have made any serious inroad—the attempt is, however, certainly worth making. If vomiting is really severe, the sufferer had better give in and rest for a time in the horizontal position, confining herself to lemon-juice and water, with the addition of small quantities of beef-tea, assisted by a little dry toast or toasted biscuit. Routine treatment by alcohol in the various forms so freely recommended by irresponsible advisers should, generally speaking, be avoided; if a stimulant is really needed, good brandy diluted with soda-water is perhaps the best form to give it in. Champagne, though often useful, induces in some cases an acid dyspepsia, which adds greatly to existing discomfort; and the same is true of porter, another popular remedy. Hot water is comforting to many, and is of decided use to some, when retching is painful and not followed by relief. When drugs are needed—and there are always a small number of cases in which they must be used—their administration had better be entrusted to the ship's doctor.

*Climate.*—Hot climates the climates with which we are now alone concerned, are conveniently classified as tropical and sub-tropical. The limits of the tropical and sub-tropical zones have of course been long ago defined by geographers; but climates, which depend on so many conditions besides those of proximity or remoteness from the equator, can only be described as roughly corresponding with the areas on the earth's surface indicated by their designations. By a tropical climate we understand a climate in which a high atmospheric temperature is maintained, with comparatively little variation, throughout the year. In these climates for about four months, spoken of as the "hot season," the thermometer ranges from 85° to 100° F. and over, during the day; while during the night there is seldom a greater fall than from 5° to 10° below this. Tropical climates are further distinguished by the rainfall, which occurs at stated periods, and is practically limited to two or three months of the year, constituting what is known as the "rainy season" or simply "the rains." In climates distinguished as sub-tropical, while several months may be cool and one or two even cold, there is during the hot season, the late spring, summer, and early autumn, such a decided and sustained rise in temperature as to make the conditions of life and the manifestations of disease at that time practically identical with those observed throughout the year in the tropics. The climates of Calcutta and Singapore are tropical; those of Cairo and Shanghai are sub-tropical.

*Effect of Heat.*—A certain number of the cases described as sunstroke and heat apoplexy may quite possibly have exposure to great heat as the sole cause at work in their production, but, apart from these, a high temperature cannot be regarded as of itself sufficient to cause acute illness. A sustained high temperature, however, distinctly influences the course of many diseases, and its effect, which is almost uniformly unfavourable in the case of Europeans, is exercised not only while the illness lasts, but also, and often to a marked degree, during the convalescence of the patient. When tropical heat is associated in the atmosphere with excessive moisture its effect is greatly intensified, the combination exercising a depressing influence on bodily functions difficult to realise unless actually experienced. Those who are already ill naturally suffer most, and in such weather patients under treatment all seem to lose ground, and the work of the nurse meets with little encouragement. It follows from this that the nurse, who has Europeans under her care, must give a good deal of time and attention to the various arrangements best calculated to lessen the injurious effect which excessive heat is sure to have on her patients.

Among the means at her disposal for this purpose the punkah, as the most generally useful, first claims our attention.

(*To be continued.*)

### Sketches from the Maratha Plague Hospital, Bombay.

By One of the Nurses.

(*Continued from page* 9.)

But to return to the wards; one is especially set apart for native treatment. On admission to the hospital the choice is given to the patient between that and English treatment. It is most amusing to see the old *Vaidya* (native doctor), with the sleeves of his jacket rolled up above the elbow, bustling from bed to bed, and roaring to each patient in turn "Jíbh cá!" or "Jíbh dákíwa!" ("Put out your tongue!") At first he did little for these patients but smear the bubos with yellow powder, and give them some medicine to drink. Now, however, he has adopted our poultices and many other English remedies.

### Water refused in the Native Ward.

The natives have a curious idea that water is injurious to those suffering from fever, so the poor creatures in the native ward are denied it. Often as we pass by they raise their eyes to us in piteous appeal and gasp "Páin" ("Water"). They cannot understand that we are not allowed to interfere with them. The strangest part of it is that the old *Vaidya* likes us to visit his ward, whilst the convalescent patients are almost as friendly as our own, being quite ready to accept fruit or biscuits from us, and even books. Yes, we have a library. It is true that it all fits very comfortably into a 7-lb. biscuit tin, but it boasts of a hundred or more of stories and essays on social or domestic subjects, in both Marathi and English. Many of the small boys can read, and are very glad to while away in this manner some of the weary hours of enforced idleness. It is amusing, when one can steal a little time, to run into the convalescents' ward, and change the books. The men are such children that they always want the biggest book. One entitled *How to bring up Children* is of quite a respectable size, and I remember having great difficulty in persuading a young lad of 16 or 17, who had set his heart on reading it, to choose another to interest him.

### Patients and Biscuits.

The Government have spared no pains in their endeavour to make the hospitals more popular. An honorary visitor specially appointed comes frequently to inquire into destitute cases, furnishing them with money to take them back to their far-away homes, and sending presents of biscuits, fruit, &c., to supplement the hospital diet. The fruit, of course, is always readily accepted; but the biscuits at first did not meet with equal favour. The patients would take them with limp fingers, look at them and look at us as if to read in our faces whether or no we meant well by them, and ask if they were to be eaten. On being assured that that was just what they were for, they would return them with a shake of the head and a most emphatic "Nako" ("Don't want"). The children, however, were wiser, and readily disposed of as many as they could persuade the "Sister Sahebs" to give them; so when the men and women saw that the *chokare lôka* (little people) enjoyed them so much, they began to eat and enjoy them equally.

### An Old Brahmin.

Two amusing stories recur to me. We had as a patient at one time an old Brahmin named Sitaram; he was evidently of a very pleasant and happy disposition, and even when seriously ill was so merry and jovial in his delirium that those around him could scarcely repress a smile. During his convalescence he developed a most enormous appetite. One day the Sister on duty was taking the biscuits round, when he demanded some. "You ought not to eat them, Sitaram," she said. "You are a Brahmin." "Never mind," replied Sitaram promptly; "English things all good." And he showed his approval by eating as many as he could get till he left the hospital.

### Breaking down Caste Prejudices.

The other story is of a Paradisi who never could be persuaded even to touch one. He was a tramcar driver, a man of splendid physique. At first he was desperately ill, and a most disobedient and troublesome patient. However, he recovered, after doctors and nurses had lost all hope, and was transferred from the acute to the convalescent ward. It was Christmas-time; a friend had sent some iced and highly-ornamented plum-cake for the convalescents. We had not supposed they would eat it, but thought that the children would be pleased with the gay flowers and birds that adorned it. Next day, however, we found that it had disappeared, and the whole ward was enjoying a joke at the Paradisi's expense. One of the men had cut up and distributed the cake, and Bhaiyá had taken a slice. "What is it made of?" he asked. They told him: "Flour, currants, sugar, eggs." "Eggs!" he returned dubiously. "I don't know if I ought to eat eggs." "But you ate eggs in Ward 1," the man replied, referring to the egg-flip given to the patient in the acute ward. "Did I?" answered Bhaiyá. "Then I may as well eat this." And he did, as well as a piece of another cake which was sent soon after. So much for caste prejudices, which are slowly but surely breaking down.

### The Children.

No description of the Maratha Hospital would be complete without some account of the children. Many of them have suffered sadly, but as a rule they recover more completely and quickly than their elders, and the little fellows in the convalescent ward are very merry, and sometimes almost riotous. Like English children, they take very kindly to spoiling, and it is to be feared that they get it in hospital. We are so proud of, and so pleased with, the comparatively few patients who recover that we can scarcely help spoiling them; and when one considers that during the month of March, 1899, alone 893 plague patients were admitted, out of which the terrible total of deaths was 734, one cannot wonder that it should be so.

### Pathetic Scenes.

Some of the children come in perfect health with their stricken parents. Father or mother or both die, and the little ones are left. Tiny dots of eight and nine, with old and anxious faces, wait assiduously upon their sick, moisten their parched lips with water, brush away the tormenting flies, and with loving words and caresses coax them to medicine or nourishment. Sometimes the Sister on duty hears a heart-broken cry—"Áí malá bola!" ("Mother, speak to me!"); or, "Májha bapa gela ahe!" ("My father has gone!")—which tells her the little one's task is finished.

When the first burst of grief is over, these children usually attach themselves to us, hover round our table, and offer to fan us, or to fetch and carry. Sometimes they present us with a stalkless flower which they have hurriedly plucked from the garden (braving the wrath of the irate *mali*) by way of expressing their gratitude. Often they devote themselves to some sick or motherless baby, who benefits greatly by their ministrations. Boys and girls are alike good as nurses; and the boys think no shame of carrying about the wee babies, kissing them, and cooing to them in a way that is really astonishing.

### Child-Wives.

Occasionally a few days after the parent's death a grown man will come to claim his child-wife. A *sagá wallá*

(relation) has written to acquaint him of her loss, and he has travelled from Poona, Ratnagiri, or some far-away *gamu* (village) to look for the girl and take her back with him to live with his mother. Then the little one clasps the hand of one of us in hers, and puts it to her forehead and her lips, and ere we are aware is down on the ground clasping our feet. Raising to ours her large dark eyes with their sweeping lashes, she cries: "Salaam! Mem Sahib caput; caput, Salaam!" ("Many, many thanks!"), and trots off behind her husband.

Little Báloo.

Very interesting was the case of four-year-old Báloo. He and his mother were both admitted suffering from plague. Very soon the mother died. Little Báloo's convalescence was tedious. He had two cervical bubos; both had been incised. The dressing was a painful process, and day and night the poor child's plaintive wail might be heard. The burden of his cry was ever, "Ái! ái!" ("Mother! mother!"). At last he began to mend. As soon as he was able to slip off his cot, he was discovered wandering anxiously from bed to bed, raising each blanket and peering at the haggard face beneath it, in the vain hope of finding his mother. Mercifully, this sad state of things soon ended. Báloo grew strong and happy again, and a merrier and a more intelligent little fellow could hardly be found. He was thus described in the following lines:—

Who is this funny little waif,
From Death's cold arms late snatched and safe?
'Tis Báloo.
Báloo, our hospital's pride and joy;
Báloo, the browniest and sauciest boy;
With a face often dirty, but never long;
With a laugh so merry it sounds like a song.
That's Báloo.

His clothing is somewhat scant, you know;
But then, I am sure, he prefers it so.
Just a tiny jacket of washed-out blue,
That has long since lost its brilliant hue;
And as often as not it is hidden away,
Whilst Báloo without it trots off to play.

His shaven crown, you must own, is neat;
And so is the shape of his dear dusty feet.
And he has the most bewitching way
Of lisping, "*In Sahib, malen biskut da!*"

Who could resist a rogue so quaint?
Not the Sisters, though they make a feint
Of keeping order and looking severe.
But Báloo knows not what it is to fear.
Life for him is one long stretch of fun,
From early morning to set of sun.

(*To be continued*)

### Private Nursing in Rome.

By a Correspondent.

"See Rome and die" is a very common expression, but it is not every nurse who has the opportunity, and many have to die without getting a glimpse of the Eternal City.

Private nursing in Rome is much the same as it is elsewhere. One has to take the rough with the smooth.

The Patients.

The patients are usually English or Americans, chiefly well-to-do, and the work lies mostly in the various hotels and pensions. The cases are generally short, but there are a few exceptions. One occasionally comes across a typhoid; but influenza and pneumonia seem to occupy most of the nurses time. I was fortunate to escape cases of "nerves," but I often heard other nurses complain of hysterical ladies they had to deal with.

A Short Season.

The nursing season in Rome is very short. The work does not, as a rule, commence before December or January, and is usually over by the end of April or the beginning of May. Of course there are exceptions to this. I heard one doctor say that he had been busier at the end of May than at the height of the season. But most of the English and American people have moved higher up by then. It is not possible for a nurse to make her way in Rome on her own account unless she has worked for the English doctors previously. They might send for her at a push, but they usually employ their own special nurses.

Knowledge of Italian Desirable.

There are two English nursing homes, where nurses are generally kept well employed by the same doctors each season. It is a great advantage for a nurse to be acquainted with either the Italian or the French language—so many of the Italians speak French too—though it is not by any means essential, as English is spoken in all the hotels and in most of the pensions.

But if a nurse finds herself placed as I was in one instance, on a large flat, with four patients down with influenza, all in different rooms, and only one ignorant Italian woman to do the cooking and cleaning, a knowledge of the language would come in useful. The poor woman had no idea of cooking, and I was obliged to prepare everything myself. I could not explain all I required for my patients, and I had to hunt for every utensil till I found it. The usual cook was one of my four patients, and this woman was hired as a make-shift. Fortunately the cook recovered first, and when the doctor pronounced her well enough to resume her duties my delight knew no bounds. This case I classed with the "rough" in my experiences, as the ladies were unable to afford a second nurse. But I learnt a great many lessons, and never regret the month spent with them.

Plenty of Going-about.

I did a great deal of sight-seeing. Often before my patients were well enough to go out, they were eager to continue their interrupted explorations. I saw a great many places of interest with them during their convalescence. Also between cases I often had two or three days to spare, and crowded in as much as possible. I took one patient and her mother to Frascati, where we stayed for six weeks. We had a most delightful cottage all to ourselves, situated on the estate of the Villa Aldo Brandisi, owned by the last surviving descendant of the ancient Borghese family. We had a peasant and his wife to do the housework and cooking. The grounds were well wooded, and we got a charming view of the blue Mediterranean in between the trees. My patient was mentally afflicted, but only occasionally; so we were out in the open air a great deal. We used to have breakfast in the garden at 7.30 a.m. Then a little pony-carriage was at the door every morning at 8.30, and we explored the country for miles round during our six weeks' stay.

A Case of Malarial Fever.

Previous to this I nursed a case at Perugia for our English doctor in Rome. There were three of our nurses at the Grand Hôtel together, one working with me. We had a girl suffering from malarial fever to nurse. Her temperature was very high, and every possible means was used to reduce it, but in vain. So the doctors decided that she must be moved carefully on to the South of France, as her temperature, they thought, would remain very high as long as she stayed in Perugia. We started for Cannes, the doctor accompanying us, doing the journey without a break in sleeping-cars. Before we reached Genoa the patient began to revive, and the temperature there had fallen from 104° to 102°. When we reached Cannes it was 99°, and in a fortnight's time the girl went for her first drive.

### Gastro-enteritis caused by the Accidental escape of Sulphuric Fumes.

By a Private Nurse.

A short time ago I was engaged to nurse a little girl who was suffering from scarlet fever. After being isolated for seven weeks, desquamation having ceased, the necessary arrangements were made by the physician with the Local Sanitary Authority for the disinfection of the three rooms, &c., which had been entirely set apart for our use in the house. Formalin was ordered for the purpose, as it had been our chief disinfectant for use during that period. A moderate-sized double-bedded room was first made ready for our reception; and, after disinfecting our persons in a complete change of garments, we vacated the other two rooms, and all was so far satisfactory.

The following day the man returned to attend to the other rooms; but, not having sufficient formalin, he substituted sulphur. The rooms were hermetically sealed just after mid-day, and the man, having finished his work, returned to his home. During luncheon there were sundry whiffs of sulphur, and before 2 p.m. the smell of it was increasing. Kneeling down to the skirting-board nearest the rooms, we found that it was stronger there; and on further nvestigation outside the rooms, I discovered a slight cloud of smoke coming from—where we could not ascertain.

It was evident that there was no time to be lost in getting help; so with addresses provided me by the lady of the house, and leaving my patient with her mother, who was happily with us, I set out at once in a cab. After some delay I found the Assistant Sanitary Inspector, and explained to him what had occurred. He volunteered to fetch the man. So, leaving the cab for the purpose, I returned to the house on foot, and found the fumes increasing in our room and in the rooms above. The men arrived five minutes later, and asking for wet cloths, which they fastened over their heads and faces, they forced the door open. We had already inhaled a fair amount of sulphur, but it became suffocating for some minutes, and to breathe we were forced to put our heads out of window, and even there we were not free, as a current of sulphurous air was pouring through a window near from the forced-open door, and the wind conveyed it to us. It was found that, the house being old, the fumes of sulphur had escaped through the minute crevices, and so filled the building. By 4 p.m. the men pronounced all safe and went away, and as we kept our window open for an hour the fumes soon left very little trace.

The next day my little patient appeared languid and ill, and her mother, being anxious, asked the physician to see her. He looked grave on hearing our description of the fumes, and ordered complete rest in bed, liquid diet every two hours, medicine four-hourly, careful watching, and other treatment if necessary. For five days the temperature was high, pulse and respiration rapid, frequent and grass-green stools with severe abdominal pains, lassitude, nausea, headache, sore throat and mouth, discoloured teeth, parched lips, loss of appetite, with irritability of temperament and a very faint trace of albumen in the urine. On the sixth and seventh days there was a decided improvement, so that on the evening of the seventh day she was ordered up for two hours. On the eighth and ninth days her condition was so much improved that, although still weak and ill, the physician advised that she should be carefully removed to her home on the tenth day. She bore the journey well, and after two weeks of gradual convalescence a marked change in her health was seen, and now she is quite well.

### Marriage of a Hospital Matron.

Miss Margaret Hutchison, Matron of the Devon and Cornwall Homœopathic Hospital, Plymouth, was married on Monday, at St. John's Church, Highbury, to Mr. Percy Siddons Hoyte, of Plymouth. Miss Hutchison received her early training at the Belvidere Fever Hospital and the Dundee Royal Infirmary. After an interval spent in private nursing, Miss Hutchison was offered and accepted the appointment, which she held up to the time of her marriage. The bride, who was given away by her father, was dressed in heliotrope silk voilé trimmed with lace over ivory satin. She wore a white felt picture hat trimmed with feathers, chiffon and orange blossom, and carried a magnificent shower bouquet, the gift of the bridegroom. The wedding presents, which numbered over a hundred, included a silver tea and coffee service from the nursing staff and a pair of entrée dishes from the committee of the Homœopathic Hospital.

### Appointments.

Lodge More Hospital, Sheffield.—Miss Amy E. Lewis has been appointed Matron. She was trained at Sheffield City Hospital, where she has since been charge nurse.

### Minor Appointments.

Borough Fever Hospital, Croydon.—Miss Helena McKinley has been appointed Sister. She was trained at Guy's Hospital for three years, and has been for eighteen months attached to Guy's Private Institution. Before entering Guy's she was nurse at the Children's Hospital, Cardington, Bedfordshire, and Charge Night Nurse at Holborn Infirmary, Mitcham.

Doncaster General Infirmary and Dispensary.—Miss Annie Wheller has been appointed Charge Nurse. She was trained at the Cancer Hospital, Brompton, and also at Doncaster Infirmary, and has since been assistant nurse and district nurse in the latter institution.

Grove Fever Hospital.—Miss Sarah Jane Angus and Miss Marion Gertrude Emerton have been appointed charge nurses. Miss Angus was trained at St. Pancras Infirmary. Miss Emerton was trained at Leicester Infirmary.

National Orthopædic Hospital, Regent's Park.—Miss Annie Laycock has been appointed Sister. She was trained at the Royal Albert Edward Infirmary, Wigan, and for sixteen months was charge nurse of diphtheria wards at Brook Hospital, Shooter's Hill. Subsequently she was at Dr. Sinclair's Gynæcological Hospital, Manchester.

Wakefield Infirmary.—Miss Ellen L. Shaw has been appointed Sister. She was trained at St. George's Infirmary, Fulham Road, where she has since been staff nurse.

### To Nurses.

We invite contributions from any of our readers, and shall be glad to pay for "Notes on News from the Nursing World," or for articles describing nursing experiences, or dealing with any nursing question from an original point of view. The minimum payment for contributions is 5s., but we welcome interesting contributions of a column, or a page, in length. It may be added that notices of entertainments, presentations, and deaths are not paid for, but of course we are always glad to receive them. All rejected manuscripts are returned in due course, and all payments for manuscripts used are made as early as possible at the beginning of each quarter.

### Echoes from the Outside World.

AN OPEN LETTER TO A HOSPITAL NURSE.

THERE are rumours that the question of the wholesale importation of Chinese labour will be raised as soon as the new Parliament meets, and that, failing the initiative by the Government, private members will introduce a Bill to put a stop to the process. But, surely, this ought not to be necessary. Last week I hinted that no consideration but price would weigh with those people who make it an inflexible rule to buy in the cheapest market. Is it vain, however, to appeal to national sentiment? Legislation directed against the admission of Chinese into this country may become an urgent necessity, but at the present time it would obviously give rise to further complications in the Far East. There is a simple mode of getting rid of the difficulty—namely, to support the laundries in which British labour is employed. The "titled and wealthy persons in Mayfair and Belgravia," who, in order to save a few shillings a week, send their clothes to be washed by Chinese, may be Imperialists by profession, but in practice they are evidently poor specimens.

It does not seem necessary to alter the law to enable women to vote at Parliamentary elections. In West Leeds a lady came up boldly to the polling booth, gave her name, and was allowed to record her vote. The explanation is that the name appeared on the register and was supposed to be that of a man. A similar thing happened in the Fareham division of Hampshire, but in this case the presiding officer at first refused the vote. The agent, however, persisted, and had his way. These incidents suggest that if women who pay rates and taxes will only take pains to conceal the identity of their sex until the polling-day they may exercise the privilege of voting without let or hindrance. That there is any urgent demand for a new Act for the enfranchisement of women, has not been shown during the contests which are now drawing to a close. At all events, our sex have had the good sense not to put the question in front when national issues of so much more importance have been at stake.

It is somewhat the fashion to speak sneeringly of Christmas plum-pudding as food, if not for babes, at least for children, and far beneath the notice of most grown-up people, who frequently partake of a small piece on December 25th "purely to oblige." Perhaps, however, this year, when the national dish will cost at least twice as much as usual, they may in consequence find that, after all, there is something not altogether disagreeable in the flavour. The housekeeper has been accustomed to pay threepence to sixpence a pound for her currants, fourpence to sixpence for her sultanas. This year prices at the present moment are vastly higher, ninepence being the lowest figure at which a pound of either fruit is obtainable, and prophets of ill omen are full of auguries that before Christmas comes this sum will be doubled. The reason for the sudden rise in prices is the failure of almost the entire crop. The yield is generally about 200,000 tons; this season it is barely 38,000, and England alone requires 50,000 between now and the middle of December. The only comfort for the poor, to whom Christmas time would not be Christmas without a piece of beef and a plum-pudding to celebrate the day, is that at present raisins are no dearer than usual, or only a trifle so, and a good deal can be done with raisins when well chopped. Simultaneously with the announcement of the higher prices comes an appeal from the Consul-General of Greece for subscriptions on behalf of those cultivators and labourers employed in Southern Greece who are likely to be in danger of starvation through the failure of the crop. I suggest that those people who always object to Christmas fare, or at least pretend to do so, shall contribute largely to the fund, out of gratitude for the fact that this year there is no need for them to partake of pudding. They can with truth plead motives of economy, and the pity it would be to eat by compulsion the share which others would gladly consume by choice.

It may interest you to know that the Lady Mayoress-elect is Miss Kathleen Haydn Green. Her mother is dead, so that upon her will devolve the honours of the Mansion House. She is the granddaughter of Joseph Haydn, well known as the author of that useful book of reference, the "Dictionary of Dates." She herself has already attained some little distinction in the literary world, for she is a contributor to several of the magazines and journals, she has written some tales for children, and published a volume of verse. I expect that Miss Green will find the demand for her writings increase exceedingly during the coming twelve months, for much additional value will probably be placed upon stories emanating from the pen of the Lady Mayoress. But she will also probably find leisure for much writing so difficult to obtain that it will be impossible for her to comply with all the demands likely to be made upon her skill.

HERE is an incident which seems to me to point to the probability of private nurses being asked before long to accompany patients to a comparatively unknown country. A friend who recently went to an eminent specialist to consult him about a serious disease affecting her eyes was very much amazed when he told her that her best chance of recovery would be to go to Central Africa. She timidly inquired if Durban, where she has friends, would not do as well, but he answered emphatically in the negative, and said that the only alternative was Egypt. The particular climate she needed was, he added, Central Africa, where she would depend upon always enjoying the uniform warmth her case requires. I am afraid that there are trifling impediments, such as expense and companionship, which will prevent her from acting upon the advice. But that it should be given is not without significance, especially as at present British Central Africa only contains a few hundred European inhabitants, and possesses but two towns of much importance, Zomba and Blantyre.

BATTLEFIELDS, Boers, and British heroes in khaki are naturally beloved by the public just now, and it is not surprising that the scene of two acts in the latest production at the Lyceum Theatre, a melodrama of regulation type with villainy and virtue introduced in regulation quantities, is laid in South Africa. "For Auld Lang Syne" is nothing if not up to date, and along with the roll of drum and the roar of battle comes the hospital nurse. Her name is Dawn Ingram. As to her training, I can tell you nothing, but both her attire and her behaviour on the field, where, presumably she is on duty, are unusual. She is "gowned" in pale lavender grey, which does not to a practical mind suggest a colour likely to look its best amidst the carnage with which she will probably come in contact, more especially as the dress is made with a very long train. Imagine the latter in a small tent with half a dozen sick men! A big white apron and a very becoming cap, a red cross on a white band upon her arm, and a crimson pincushion hanging from her waist complete the costume. The Boer bullets are whizzing around, and the men are dropping on every side; but "Nurse Dawn," with apparently no qualms of conscience, allows Lord Fellsdale to make love to her as the battle proceeds, and the high-flown sentiments of the absorbed pair barely reach the audience above the din of musketry. The play is pronounced "very realistic"—and I must say the scenery is beautiful—but the nurse belongs entirely to the realms of romance.

### Everybody's Opinion.

[Correspondence on all subjects is invited, but we cannot in any way be responsible for the opinions expressed by our correspondents. No communication can be entertained if the name and address of the correspondent is not given, as a guarantee of good faith but not necessarily for publication, or unless one side of the paper only is written on.]

### VISITING NURSES FOR THE MIDDLE CLASSES.

The late matron of the Dorset County Home for Nurses at Dorchester writes: *Re* your article "Visiting Nurses for the Middle Classes" I should like to tell you that the principle is being successfully carried out by the Dorset County Home for Nurses, Dorchester. There is a subscription list, which enables the committee to send nurses to those who cannot afford to pay the usual fees, one guinea to 5s. per week being charged, according to the patient's circumstances, but the majority of cases are 10s. 6d. per week. Board and lodging are very often given to the nurse at the house free; and when they are nursing in the country cottages someone comes forward, in nearly every case, to offer the nurse accommodation. The matron first receives the application for a nurse, and requires a note of recommendation from the attending doctor, a subscriber, or a minister of religion, on receipt of which the nurse is sent; but each case is eventually brought forward at the following committee meeting. The nurses are all fully trained, and nurses those who can afford the full fees as well. The maternity nurses especially are much appreciated, and by the wives of mechanics, clerks, &c., who cannot share the district nurses, and so usually have the untrained so-called "Gamps." There is very little demand for daily visits among any class, although we advertised that such could be given.

"One of the Public" writes: I was extremely interested to see the article on this subject in last week's Hospital, for it has been brought before me vividly lately how very much nurses are wanted for a class of person who *cannot* pay high fees. I see visiting nurses advertising their services at 3s. 6d. an hour; but this is, of course, a prohibitive price for any but the well-to-do to pay. It means that a person who has the services of a visiting nurse daily pays at the rate of almost 25s. a week for services of an hour a day! Is not this just a little preposterous, when other nurses give their whole time for £2 2s. a week? The people who need visiting nurses most are: first, the middle-class gentlefolks whose means are limited; second, small tradespeople; and third (and it is this latter class for whom I am especially anxious to plead), the large number of working ladies who live in our big towns—daily governesses, secretaries, typewriters, and others *ad infinitum*. They usually live in one or two rooms, or in small flats. When they are ill their condition is one of great wretchedness. They do not like to go into the general ward of a hospital, they have no one to look after them, and they cannot afford to pay for a nurse's whole time. Very often they are not so ill as to require more than a daily visit, or two daily visits; but if these could be arranged at *moderate* fees, it would be an inestimable boon. The fees must, however, be really moderate, otherwise nothing is gained. In fact, what we want is district nursing for a class above the actual poor, but which is often struggling with as great poverty as the poorest. A case of the kind has come before me lately. A poor governess was ill and needed someone to sit up with her for one night. The charge made by the nurse for doing this was £1 1s., including her cab fare! The same governess asked if a nurse could go to her for an *hour* one evening—she was charged 5s. 6d. for the nurse's visit; and as she is living literally from hand to mouth, this was a very serious pull on her resources. I believe the want for visiting nurses at low fees is a real one, and there seems no reason why, if a visiting nurse worked over a certain area as a district nurse does, she should not be able to make it pay without an undue expenditure of strength. It seems as though it would at any rate be worth while to ventilate the subject and review its possibilities.

### NURSES' READING SOCIETY.

Miss Moberly writes: I am sorry to say that there has been a considerable falling-off of members lately, or rather I should say that members have simply left me in the lurch—neither paying their subscriptions, nor having the courtesy to inform me of any wish to leave the Society. Unless old members pay their subscriptions, or we are reinforced by new members, I fear the Society will not be able to exist after the end of next July, when the year ends. Will nurses belonging to the *library kindly always inform Mr. Glaisher what book they require?* As there are two books being read each quarter, he naturally does not know which one to send to individual nurses unless they tell him.

### ACCIDENTS TO CYCLISTS.

"E. S." writes: "Why do you not, in your valuable paper, advise all nurses to insure against accidents? I am a private nurse, and insure against accidents of all kinds and several fevers. This summer I met with a bicycle accident, and received £20 from an insurance company. They are most fair and punctual in their payments."

### NURSING ON THE "ASSAYE."

The Manager of the A.M.B. Relief Corps writes: "In your issue of September 29th, in an article on 'Nursing on the "Assaye,"' Sister Millar, in regard to giving out clothing and tobacco, says: 'The things were brought on board by Professor Dunlop, and were mostly given by the ladies of Coatbridge and Airdrie in Scotland.' The Tommies on this voyage must have been wonderfully comfortable if the ladies of Coatbridge and Airdrie supplied 'most' of the things. For this voyage of the 'Assaye'—delivered to and signed for by Dr. Harvey, the P.M.O. in charge—the contribution (on our regular scale) of the Absent-minded Beggar Relief Corps was as follows: 350 complete outfits (6 articles in each outfit), 900 shirts, 900 pairs socks, 900 caps, 900 handkerchiefs, 24 pairs slippers, 113 pillows and cushions, 6 pairs crutches, 12 sticks, 1,250 pipes, 297 lbs. tobacco, 1,200 packets cigarettes, 120 packs of playing-cards."

### LECTURES BY "PRO.'S."

"A Lancashire Lassie" writes: I am very interested in reading the various letters sent in to your columns. One gains so much by the hints offered. Having had a little experience of nursing and convalescent work myself, and hoping to still go in for general training, it has set me thinking lately how hard it is for some of the "Pro.'s" who are not very bright at mental work—but may be *vice versâ* in practical work—to successfully pass their exams. Once a week, as a rule, the doctor or matron gives them a lecture, which they are supposed to take down in notes, but unless one knows shorthand is no easy thing to do, as the doctor often talks rapidly and difficult words keep occurring, which make one hesitate before writing them down, and by the time one has written them the doctor is far ahead, and it is necessary to take a bound to catch him up. Do you not think it would be a good idea for each "Pro." to take it in turns to give lectures to the others on whatever subject she has been studying or is interested in—of course I do not mean to suggest the omission of the doctor's lectures—and for the others to criticise and add to if they know more on the subject? A little time once a week spent in this way—say the day when no operations are on—might benefit the probationers, and also the institution to which "they are attached."

### A Book and its Story.

### MR. MERRIMAN'S NEW ROMANCE.*

A WILD land and a wild people, the country of the Mistral, of mountain passes and mountain strongholds where the bandit and outlaw hides, and no native moves abroad without a gun or pistol for his own protection or his neighbour's destruction; where the roadside is strewn thick with little crosses, each marking the grave of those who have fallen by the way without their murderers being brought to justice. For justice is difficult and tardy in this half-barbarous land, in spite of its being an island in the Mediterranean, within reach of civilisation of a high order.

In this so-called "Island of Unrest," otherwise Corsica, the chief incidents in Mr. Seton Merriman's latest romance centre. The good old-fashioned elements of romance are here. There is love, of course, and war; there are plots and counterplots. There is a villain of the gentlemanly order; a heroine with character; her lover, who is a soldier and patriot; a village *curé*; and an old maid—no insignificant person and very necessary to the background, in which she moves with decision. There is also a fascinating little Baroness de Mélide, whose husband, the Baron, "was a large man with a vacant face which concealed a very mine of common-sense," and various other persons of more or less distinction.

The story opens in the spring of 1870. Corsica was watching events. Disturbance in the country to which she was tributary meant a possible opportunity of rising and shaking off its yoke; for it was only because Napoleon III. was a Buonaparte that Corsicans endured his rule—a rule which in its iron tyranny suited them better on the whole than any more tolerant, for they are born insurgents, and require firm, unyielding handling.

The Emperor on the eve of the Franco-German War is thus written of:

"It will be remembered that the third Napoleon—the last of that strange dynasty—raised himself to the Imperial throne—made himself, indeed, the most powerful monarch in Europe—by statecraft, not by the power of the sword; . . . perhaps he had no taste for Ministry or bureau, nor cared to cultivate the subtle knowledge of Court and Cabinet which meant so much at that time. . . . His tastes were rather those of the camp; and, failing war, he had turned his thoughts to sport. He had hunted in England and fished in Norway, and shot big game in Africa."

In the spring of 1870 he returned

"to find France and his dear Paris gayer, more *insouciant*, more brilliant than ever. For the Empire had never stood higher in the eyes of the world, nor seemed more secure, than it did at this moment, had never boasted a more lavish Court."

"No one knows why in July of the same year Napoleon III. declared war against Germany. The secret of the greatest war of modern times lies buried in the Imperial mausoleum at Frognal. . . . If France was not ready, she thought herself so. . . . She was so eager that she shouted when she should have held her tongue. And who shall say what the schemer of the Tuileries thought of it behind that pleasant smile, those dull sphinx-like eyes? . . . He was never a flustered man, this nephew of the greatest genius the world has seen . . . and he made his preparations quietly."

Lory de Vasselot, a young cavalry officer who distinguishes himself in war, has property in Corsica. The orphan heroine, Denise Lange, educated under the charge of Mademoiselle Brun, becomes mathematical mistress in the convent school of The Sacred Heart in Paris. Mademoiselle Brun herself had, by dint of pinching and saving, managed to educate herself, and maintain Denise, in a manner peculiar to her sex and nation.

"For French women are not only cleverer and more capable than French men, but they are cleverer and more capable than any other women in the world. History, moreover, will prove this; for nearly all the great women that the world has seen have been produced by France."

One fine day Denise, sitting alone with Mademoiselle Brun in their dull little apartment, is handed by the cross-grained old nun who acted as their concierge an official-looking envelope.

"'It is addressed in a man's handwriting,' she said, warningly.

"'Then let us by all means send for the tongs,' answered Denise.

"She looked at it curiously before she opened it, and glanced towards Mademoiselle Brun. It was, perhaps, characteristic of the old schoolmistress to show no interest whatever. And yet to her it seemed an age before the girl came towards her with the open letter in her hand. . . . She gave a joyous laugh, and threw the letter down on Mademoiselle Brun's knees.

"'It is a romance inside the blue envelope—that is all.'

"'It is my father's cousin, Mattei Perucca, who has died suddenly, and left me his estate in Corsica. . . . Here is the address, where I shall invite you to stay with me.'"

Baroness de Mélide decided, on hearing of Denise's good fortune, that she must marry Lory de Vasselot, and writes at once to Mademoiselle Brun:

"'From my dear good cousin, Lory de Vasselot, who is, if you will believe it, a Corsican neighbour, I have already heard of Denise's good luck. The Vasselot and Perucca estates actually join. Both, I need hardly tell you, bristle with bandits, and are quite impossible. I have quite decided that Lory shall marry Denise. I need not tell you to see that Denise looks pretty; the good God will see to that. And as for Lory, he is an angel.'"

The two meet at the Baroness's and discuss the situation. Denise in her ignorance of the world, and especially of the particular part of it in which her estate lays, declares her intention of going to Corsica to inspect and, if possible, live on it.

"'A woman! you are not going to Corsica, Mademoiselle?'

"'But I am,' she answered.

"De Vasselot spread his hands in utter despair. . . .

"Denise only laughed.

"'And when I have regulated my own affairs, I will undertake the management of your estate at a high salary,' she said."

By which suggestion and intention it will be seen that Denise was a young woman with a will of her own. To Corsica she goes, and so does Lory de Vasselot, who is recalled almost immediately by the outbreak of the war. Enough has been said to indicate the outlines of this cleverly-written story, into which Mr. Seton Merriman has put, as is usual with him, much vivid scenic description, graphic characterisation, and epigrammatic dialogue. Few living writers excel more in the art of vignette writing; each character, each scene, every conversation carries with it its own quiet force, by absolute simplicity and fitness to the occasion in unemotional scenes, and by the dramatic power shown in others, where deeper currents are stirred. No one, perhaps, objects more than an author of recognised standing to be presented to the public in paragraphs, but we would in conclusion make one or two extracts to illustrate the literary points emphasised.

"The proudest man is he who is sufficient for himself."

"The French—the most intellectually subtle people in the world—have a certain odd simplicity which seems to have survived all the changes and chances of Monarchy, Republic, and Empire."

"A Frenchman is not nearly so artificial as the shallow British observer has been pleased to conclude. He is, in fact, much more a child of nature than either an Englishman or a German."

"Life is full of gravity, and the gravest part of it is love."

* "The Isle of Unrest." By H. Seton Merriman. Publishers: Smith Elder and Co. Price 6s.

### For Reading to the Sick.

DEATH is the veil which those who live call Life ;
They sleep—and it is lifted !—*Shelley.*

Sweet Saviour, in mine hour of mortal anguish,
When earth grows dim, and round me falls the night,
Oh, breathe Thy peace as flesh and spirit languish ;
At that dread eventide let there be light.

. . . . . . . .

To Thy dear Cross turn Thou mine eyes in dying ;
Lay but my fainting head upon Thy Breast ;
Those outstretched Arms receive my latest sighing ;
And then, oh then, Thine everlasting Rest.
*Hymns A. and M.*, 121 (E. ALDERSON).

**Reading.**

"SUFFER us not from any pains of death at our last hour to fall from Thee."

THE saintly Bishop Hall writes of his entry into rest :—

O death, how sweet is the rest wherewith thou refreshest the weary pilgrims of this Vale of Mortality !
How pleasant is thy face to those eyes that have acquainted themselves with the sight of it ! . . . .
How worthy art thou to be welcome unto those that know whence thou art, and whither tendest !
Who that knows can fear thee ?
Alas ! O my God, nature is strong and weak in me at once ! I cannot wish to welcome death as it is worthy ; when I look for most courage I find strongest temptations ; I see and confess that when I am myself Thou hast no such coward as I.
O Thou God of Spirits, Thou hast coupled body and spirit together ;
Unite them in a desire of their dissolution ;
Weaken this flesh to receive and encourage this spirit either to desire or to contemn death ; and as I grow nearer to my home let me increase in the sense of my joys.
I am Thine ; save me, O Lord.

. . . . . . . .

This body shall not be buried, but sown,
And at our day shall therefore spring up
With a plentiful increase of glory.
How comfortless, how desperate
Would be our lying down
Were it not for the assurance of our uprising !
The power that can raise one man can raise a thousand, a million, a world ;
No power can raise one man but that which is Infinite ; and that which is Infinite admits of no limitations.
It is sown in corruption, it is raised in corruption ; it is sown in dishonour, it is raised in glory ; it is sown in weakness, it is raised in power. (1 Cor. xv. 43).
And when thou raisest thine eyes to Heaven think of that glorious society of blessed Saints who are gone before thee. Bless God for them. Tread in their holy steps and wish thyself with them.—*Meditations* (BISHOP HALL).

O Christ the Life, look on me where I lie
Ready to die :
O Good Samaritan, nay, pass not by.

O Christ, my Life, pour in Thine oil and wine
To keep me Thine ;
Me ever Thine, and Thee for ever mine.

Watch by Thy saints and sinners, watch by all
Thy great and small ;
Once Thou didst call us all—O Lord, recall.

Think how Thy saints love sinners, how they pray
And hope alway,
And thereby grow more like Thee day by day.

O Saint of Saints, if those with prayer and vow

Succour us now . . .

It was not they died for us, it was Thou.

*C. Rossetti.*

### Notes and Queries.

**The Editor is always willing to answer in this column, without any fee, all reasonable questions, as soon as possible.**
**But the following rules must be carefully observed :—**
**1. Every communication must be accompanied by the name and address of the writer.**
**2. The question must always bear upon nursing, directly or indirectly.**
**If an answer is required by letter a fee of half-a-crown must be enclosed with the note containing the inquiry.**

*Training.*

(22) 1. Where can I train to become a nurse ? I am 21 years of age and want to start at once. 2. Are probationers taken at Netley Hospital ?—*Katie B.*

1. See the "Nursing Profession ; How and Where to Train." 2. No ; only fully trained ladies.

*Inebriates.*

(23) Is there any home or hospital for inebriates where a good laundress could give her services in return for treatment ? The woman is poor and anxious to be cured.—*Nurse Leila L.*

The Diocesan C.E.T.S. Home for Middle and Working-class Women, Liverpool, receives some free cases. Apply to Miss Agnes Wilson, Woman's Temperance Home, 318 Upper Parliament Street. See other homes in the "Englishwoman's Year Book."

*Plague Nursing.*

(24) Kindly tell me where I can obtain information respecting plague nursing, and if two years' fever training would be sufficient qualification ?—*Topsy.*

You can get full particulars respecting plague nursing from the Revenue Secretary, the India Office, S.W. Nurses must be fully qualified by a three-years certificate from a general hospital.

*How to Apply for Training.*

(25) 1. Is it advisable, when writing to enter a hospital as probationer, to address the matron or the house surgeon ? 2. Where are "Guy's," "Bart's," "St. Thomas's," "St. James's," "St. Mary-le-Bone" situated exactly ? 3. Do all give general training ?—*E. H.*

1. The matron always arranges matters appertaining to the nursing staff. 2. St. Bartholomew's, West Smithfield, E.C. ; Guy's Hospital, St. Thomas' Street, Borough, S.E. ; St. Thomas's Hospital, Westminster Bridge Road, S.E. ; St. James's (insufficiently described) ; St. Mary-le-Bone Infirmary, St. Charles Square, North Kensington, W. 3. Yes.

*Army Nursing Service.*

(26) Will you kindly tell me how and where to apply for a Sister's post in the Army Nursing Service ?—*Sister Hilda.*

Apply to the Secretary, the Army Nursing Service, 18 Victoria Street, Westminster, S.W., from whom you will receive forms of application and instructions.

*Appointment.*

(27) Would you be so kind as to inform two hospital-trained nurses the best way of getting appointments together in either an American or an Indian hospital ?—*Flo.*

The best appointments abroad frequently are secured through large London associations. The Colonial Nursing Association, Imperial Institute, S.W., has a large connection with English hospitals in the Colonies and settlements.

*Army Nursing Reserve.*

(28) I am anxious to go to China as an army nurse. Will you kindly tell me where to write ?—*E. W.*

Apply to the Hon. Secretary, the Army Nursing Reserve, 18 Victoria Street, Westminster, S.W. If you are elected as a member of the Society, volunteer for active service.

*Medicine.*

(29) Which is the best way to set about studying medicine ?—*Janet G.*

See the Medical Section in the "Englishwoman's Year Book" for 1900 (Adam & Charles Black, publishers, price 2s. 6d.)

*Re Certificate.*

(30) Will you kindly tell me if it is lawful for a Matron to withhold a nurse's one year's certificate (after promising it), and also if it will prevent the nurse entering another hospital ?—*Anxious One.*

A one year's certificate is not of much value. A matron is under no compulsion, however, to grant it. You will certainly be able to get nursing work in another institution if fitted for it. Try for a three years' certificate in one of the large infirmaries.

*Mental Nursing.*

(31) Please inform me where I can obtain a list of County Lunatic Asylums, and how to enter one as a probationer.—*E. H.*

You will find it in "Burdett's Official Nursing Directory" under "Training Schools for Attendants on the Insane." Apply to the Lady Superintendent of Nurses.

### Standard Books of Reference.

"The Nursing Profession : How and Where to Train." 2s. net ; post free, 2s. 4d.
"The Nurses' Dictionary of Medical Terms." 2s.
"Burdett's Series of Nursing Text-Books." 1s. each.
"A Handbook for Nurses." (Illustrated.) 5s.
"Nursing : Its Theory and Practice." New Edition. 3s. 6d.
"Helps in Sickness and to Health." Fifteenth Thousand. 5s.
"The Physiological Feeding of Infants." 1s.
"The Physiological Nursery Chart." 1s. ; post free, 1s. 3d.

All these are published by THE SCIENTIFIC PRESS, Ltd., and may be obtained through any bookseller or direct from the publishers, 28 and 29 Southampton Street, London, W.C.

